# Atypical Dermal Findings in a Patient Following a Lightning Strike Injury

**DOI:** 10.7759/cureus.49096

**Published:** 2023-11-20

**Authors:** Kyle Aldridge, Kevin E Guzman, Yarelis Machin, Ilya Fonarov, Damian Casadesus

**Affiliations:** 1 Medicine, St. George's University, True Blue, GRD; 2 Internal Medicine, Jackson Memorial Hospital, Miami, USA; 3 Medicine, American University of the Caribbean, Cupecoy, SXM

**Keywords:** myalgia, lightning, weather conditions, lichtenberg figure, lightning injury

## Abstract

Lightning is a common atmospheric occurrence. However, lightning strikes are not a frequent environmental cause of human injury. Survivors may present with Lichtenberg figures, a fern-like skin manifestation, and burns of varying severity. After a lightning strike, our patient demonstrated atypical cutaneous manifestations of large, ecchymotic discolorations on the medial upper extremities. After a comprehensive evaluation, the patient fully recovered and was discharged home without limitations. This case highlights lightning strike injury, including common findings, epidemiology, mechanisms, and prevention.

## Introduction

A lightning strike injury can result in a wide range of pathologies, such as tissue destruction, and cardiorespiratory complications resulting in death. Lighting strikes are responsible for up to 300 injuries and 100 deaths annually in the United States. Lightning current can be transmitted directly and indirectly through different mediums, causing injury in various ways [1]. Our patient sustained a lightning strike injury while repairing outdoor water pipes.

## Case presentation

A male in his 60s, untreated for any chronic medical conditions, presented to the emergency department with a chief complaint of bilateral upper extremity pain and stiffness. He stated that he had been struck by lightning three days prior. The patient works in public utilities, repairing water supply pipes, and was outside during an afternoon thunderstorm. The patient was repairing a large metal pipe while ankle-deep in water. He was wearing high-top, rubber, waterproof boots, and carrying a large metal wrench in his left hand.

At the time of the incident, the patient describes feeling a sharp pain in his left bicep spreading to his right bicep along with a blinding light and loud noise. He reports stumbling forward but did not fall or lose consciousness. He denied chest pain, palpitations, difficulty breathing, confusion, deafness, or vision changes. The patient was able to continue his work. He did not experience bothersome symptoms until three days later.

On presenting to the emergency room, the patient's main concern was bilateral upper extremity pain rated 7 out of 10. The pain had been steadily increasing in severity since the day prior. He also endorsed weakness, stiffness, and a cramping sensation upon upper extremity exertion. The patient had no other complaints or concerns.

His vital signs were stable with a temperature of 36.7°C, heart rate of 68 beats per minute, respiratory rate of 16 breaths per minute, blood pressure of 124/80 mmHg, and oxygen saturation of 96% on room air. Upon physical examination, the patient was alert, oriented, and well-appearing. There were no overt features of trauma. The head, eyes, ears, nose, and throat exam was normal. The cardiac and respiratory exams were unremarkable.

**Figure 1:**
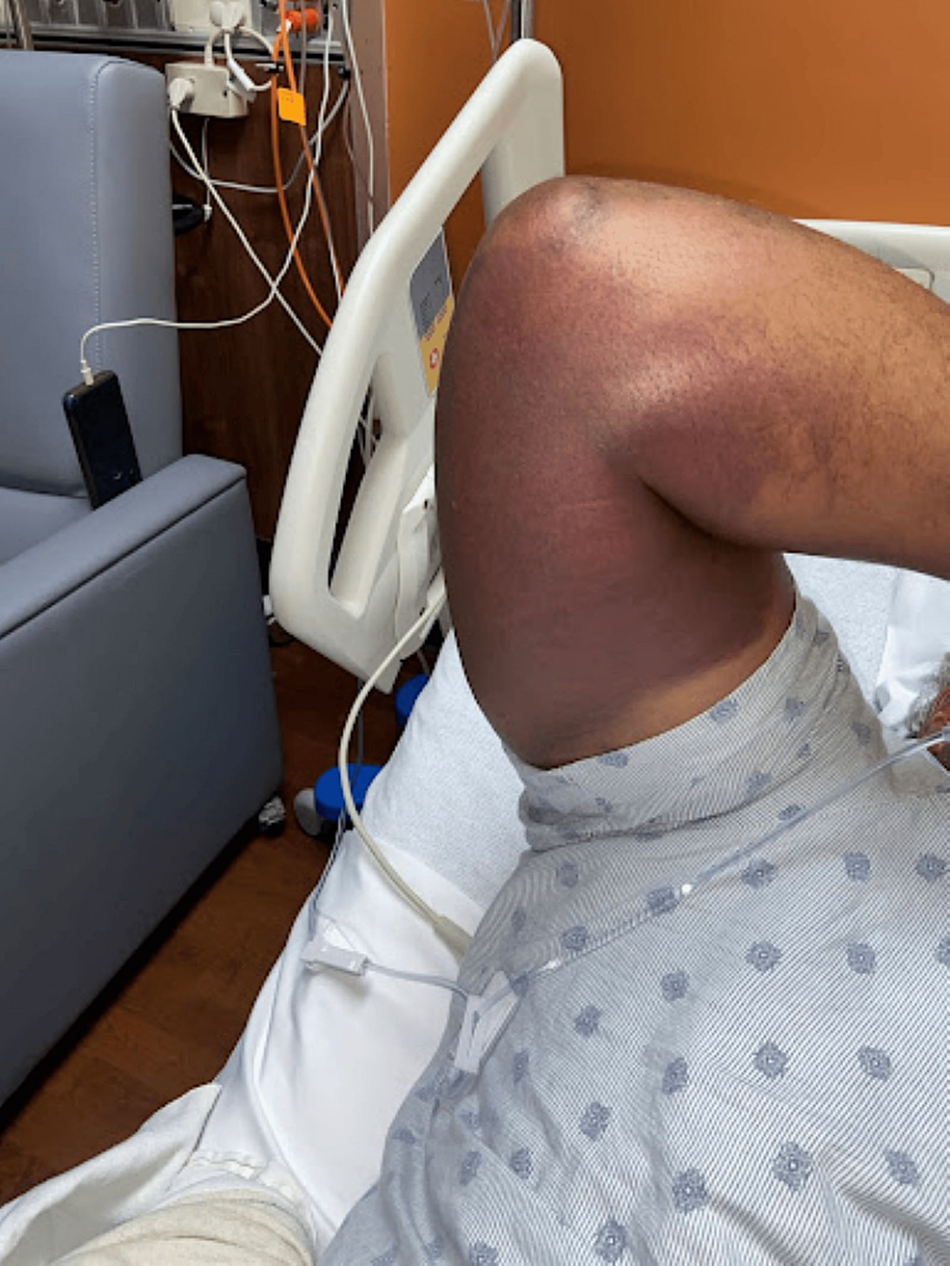
Ecchymotic skin changes on the right medial upper extremity

**Figure 2:**
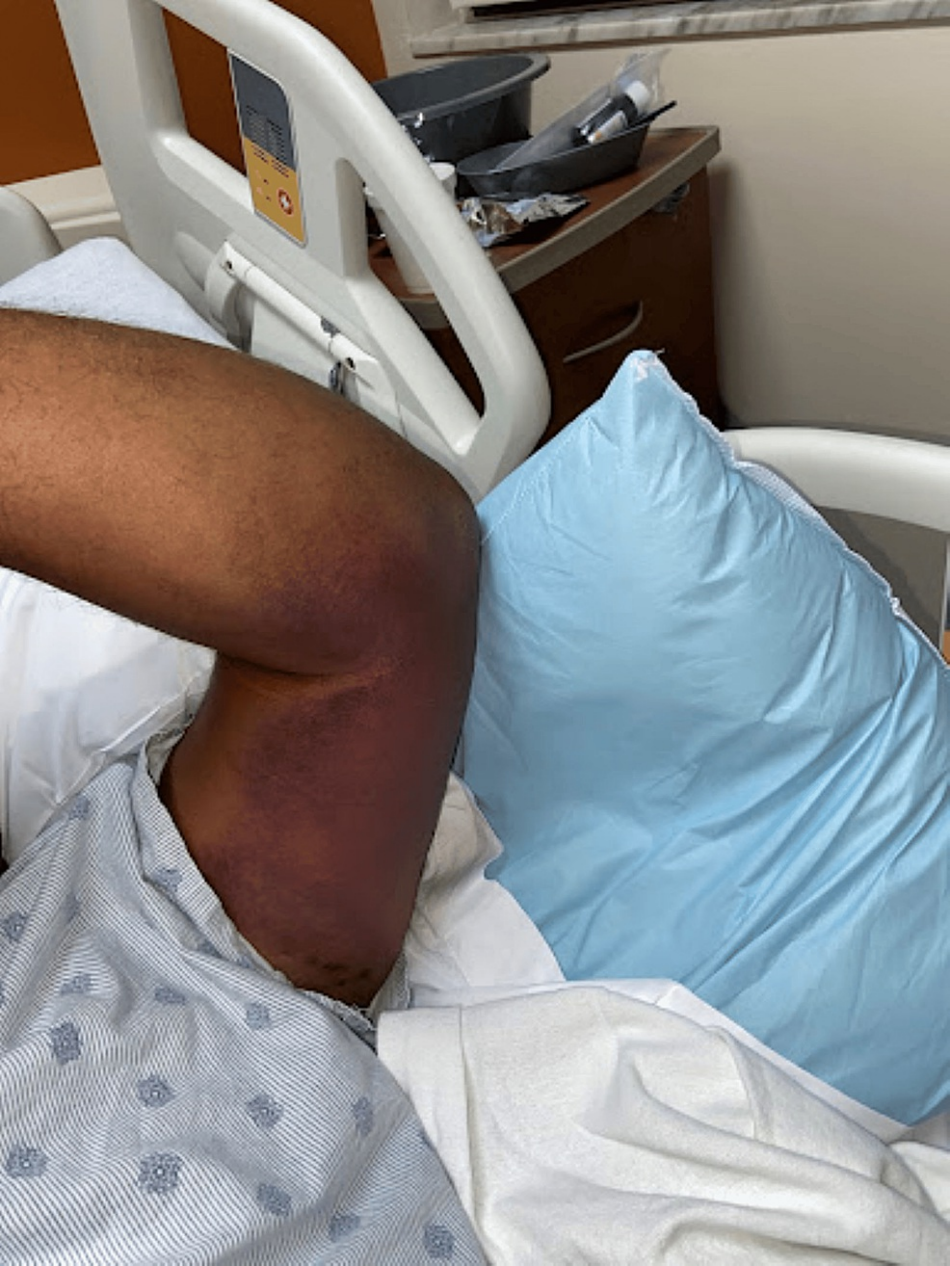
Ecchymotic skin changes on the left medial upper extremity

There were large, warm, non-tender discolorations on the medial aspect of the right upper extremity (Figure 1) and left upper extremity (Figure 2). There were no other lesions on his body. Bilateral upper extremity strength was graded at 4+/5+ with 1/4 tone. The radial artery was palpable at 3+/4+, and his hands were warm and well-perfused. There were no sensory changes on dull and sharp testing bilaterally.

He was admitted for overnight observation and further workup. The patient’s pertinent lab values can be found in Table 1.

**Table 1:** Pertinent laboratory findings in our patient

Investigation	Patient values	Reference values
Hemoglobin	12.6 g/dl	13-17 g/dl
White blood cells	6.3 × 10^3^/μl	4-10 × 10^3^/μl
Platelet count	228 x × 10^3^/μl	150-400 × 10^3^/μl
Creatinine phosphokinase	165 u/L	55-170 u/L
Troponin I	<0.012 ng/mL	<0.04 ng/mL
Urine blood	Negative	Negative
Urine protein	Negative	Negative

**Figure 3:**
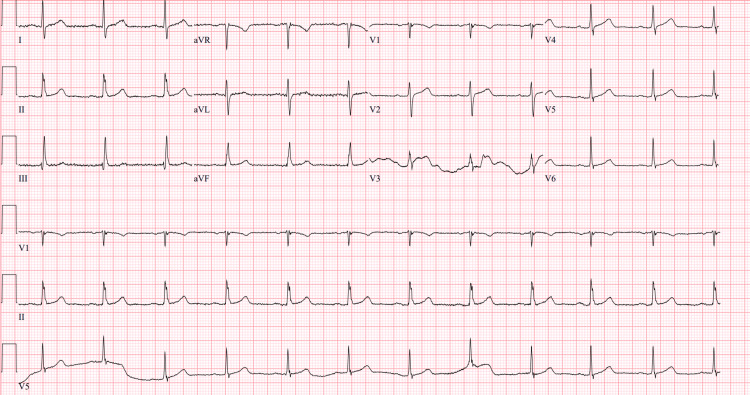
Normal ECG findings in our patient

A 12-lead electrocardiogram (ECG) showed normal sinus rhythm with no evidence of ST changes, T-wave abnormalities, or arrhythmias (Figure 3).

**Figure 4:**
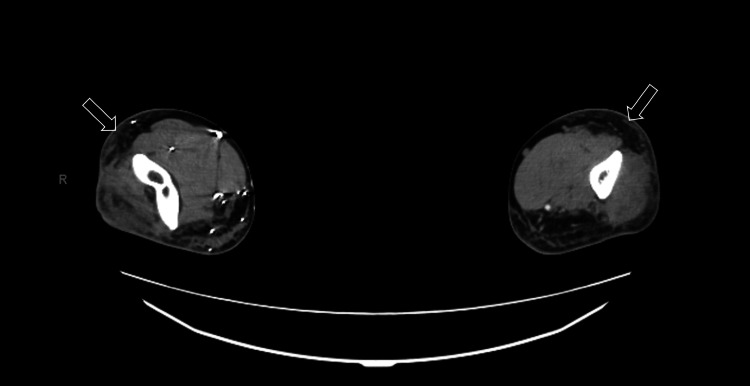
CT angiogram with contrast of upper bilateral extremities

A computed tomography angiography (CTA) upper extremity bilateral with contrast showed diffuse subcutaneous inflammatory changes, without fluid or gaseous infiltration (Figure 4).

His pain was controlled with oxycodone-acetaminophen 5-325 mg, and he received 0.45% sodium chloride intravenous solution at 125 ml/hr overnight.

The next morning, he reported no pain and showed mildly improved stiffness and weakness. His physical findings were unchanged. The patient was discharged home with supportive instructions, including acetaminophen for pain control, range of motion exercises for his mild bilateral stiffness, skin monitoring for infection, and follow-up with his primary care provider.

## Discussion

Lightning is a common and powerful atmospheric event that reaches extreme temperatures and speeds [2,3].

Lightning strike injury occurs during leisurely outdoor activities in 60% of cases [4]. Males are struck by lightning five times more frequently than females. Although 70% of lightning strikes are nonfatal, 75% of survivors experience some form of chronic morbidity after the event [5]. Patients struck by lightning can experience a marked spectrum of symptoms and findings. The injuries caused by lightning can be divided into damage from light, heat, electricity, and barotrauma. The range of damage caused by lightning can also be attributed to the different ways that lightning can strike [6].

The direct strike occurs when lightning squarely hits the victim. Entry and exit wounds are usually observed. This strike makes up 3-5% of total lightning strikes but has the highest mortality rate. Contact injury happens when lightning strikes an object that the victim is touching. Side-flash injuries arise when lightning strikes an object and then bounces over to the victim. Ground strikes are the most common and develop when lightning strikes the soil and the current flows up one leg and down the other. Injury can also occur via a blast wave where explosive energy caused by a lightning strike can throw the victim or induce trauma via projectiles. The last mechanism of lightning-induced injury is rarely seen nowadays. Phone electrocution takes place when lightning strikes a telephone line and electrical energy surges through wires to enter the handset of a held, wired phone [7].

Regarding cutaneous manifestations of lightning strikes, Lichtenberg figures are likely the most recognized. These figures are fern-like, occur rapidly after the event, and fade within hours. The mechanism behind their presentation is not fully understood. They are not burns, do not follow nerve or vascular pathways, and are not always associated with damage to the underlying tissue [8]. Burns of multiple varieties and severities are also observed. Lightning injuries can also affect the cardiovascular system. Ventricular arrhythmias, asystole, and transient ECG changes can be found. Tympanic membrane rupture and vitreous hemorrhage are also clinical features. Other considerations for the lightning-struck patient include blunt force trauma, bone fracture, and rhabdomyolysis [9].

Particular attention should be paid to rhabdomyolysis during the evaluation of lightning strike injury. This pathology has a classic, albeit rare, triad of myalgia, weakness, and tea-colored urine. Nonspecific systemic manifestations like malaise, tachycardia, and fever can be observed. Diagnosis requires an index of suspicion and measurement of serum creatinine phosphokinase (CPK). This enzyme at a level of five times the upper limit of normal is commonly used as a threshold for diagnosis. CPK can also be used to predict the likelihood of complications. Serum levels greater than 5,000 IU/L are associated with the development of kidney injury and disseminated intravascular coagulation and multiorgan failure as later consequences [10].

Injuries to the nervous system are also common, including loss of consciousness, seizures, peripheral nerve damage, and keraunoparalysis [9]. Keraunoparalysis is a unique finding after a lightning strike that usually manifests as transient paralysis of a limb with associated vascular findings like pallor and pulselessness. Up to 80% of patients experience this phenomenon. Full recovery, often within hours, is typical, and chronic deficits should be further investigated [11].

Our patient did not experience any of the severe injuries associated with lightning strikes. His presentation was atypical to those commonly found on the physical exam of strike victims. Based on the patient's report, he was crouched on a wet surface while repairing steel pipes with a wrench at the time he was struck. Given the patient's story, we considered that most likely his injury was a contact strike. He did not experience neurological, respiratory, or cardiac deficits at the time of the injury. Instead, he later presented with stiffness and weakness on exertion and bilateral large discolorations on the upper extremities.

Upon evaluation, three days after the initial strike. The patient reported pain, stiffness, and weakness in the bilateral upper extremities. His symptoms also now involved a cramping sensation on activity. His strength was graded at 4+ out of 5+, as he noted he was not as strong as usual and was unable to abduct the biceps against full resistance. On examination of tone, there was mild resistance to passive movement and this was graded as 1 out of 5. Following overnight observation and pain management as needed, the patient's symptoms subsided. However, the upper extremity discoloration remained the same intensity throughout his hospital stay. We entertained cellulitis, electrical burn, traumatic ecchymosis, and lightning-induced vasculitis as differential diagnoses for the lesions.

Our patient experienced myalgia and a CPK at the upper limit of normal indicating some degree of myolysis. Given his delayed presentation and the 36-hour half-life of CPK in serum [10], we concluded that he was not at risk for complications of rhabdomyolysis. In addition, his urine analysis was negative and an ECG was without any concerning findings. He was found to have no serious complications following the injury and was discharged home after one day of observation and testing.

Most lightning strikes occur during outdoor activities. It is of the utmost importance that these activities, including trades with outdoor labor, have a lightning-safe plan. Lightning-safe policies are proven to reduce the risk of injury from lightning strikes [12]. There are many components to such a policy. A catchy slogan to educate the public on the dangers that lightning can pose along with a chain of command for weather-related decisions must be present. Safe locations should also be designated. Fully enclosed buildings are the ideal choice. If not available, vehicles with solid metal roofs are almost equivalent. Specific criteria must be met for the outdoor activity to resume. The “all clear” may be given when lightning has not been detected within 15 miles for at least 30 minutes [13].

## Conclusions

We presented a patient who experienced a lightning strike injury. The patient reported bilateral upper extremity pain, weakness, and stiffness. Laboratory and ECG testing revealed no abnormal findings. Diffuse subcutaneous inflammation was noted on CT angiography of the bilateral upper extremities. The patient's symptoms resolved without additional complications, and he was discharged home safely. Lightning strikes are an uncommon source of injury and can present in a multitude of ways. The majority of lightning strikes are nonfatal, but chronic morbidity is noted in most patients. Prevention of lightning exposure is the most important step in the reduction of this form of injury.
